# Current concepts on anti-Phospholipase A2 receptor antibody in Idiopathic membranous nephropathy

**DOI:** 10.12861/jrip.2013.23

**Published:** 2013-06-01

**Authors:** Mohammad Reza Ardalan

**Affiliations:** ^1^Chronic Kidney Disease Research Center, Tabriz University of Medical Sciences, Tabriz, Iran

**Keywords:** Membranous nephropathy, Nephrotic syndrome, Phospholipase A2 receptor, Anti-PLA2R antibody

**Implication for health policy/practice/research/medical education:**

Phospholipase A2 receptor, is normally expressed in podocyte membrane. In fact the phospholipase A2 receptor (PLA2R) is a type I transmembrane glycoprotein related to the C-type animal lectin family such as the mannose receptor. PLA2R regulates a number of biological responses produced by secretory phospholipase A2s (sPLA2s). Determining anti-PLA2r serum levels in patients with nephrotic syndrome should designate a probable diagnosis of idiopathic membranous nephropathy and in patients in which idiopathic membranous nephropathy had a pathology confirmation may be a determining factor to exclude secondary forms of the disease. However, it is too early to abandon a kidney biopsy in patients with nephritic syndrome, and PLA2R as the newly introduced serologic study needs confirmations to finds its proper place in the field of clinical medicine.

The most common cause of nephrotic syndrome in adults is idiopathic membranous nephropathy (iMN), which is an antibody-mediated autoimmune glomerular disease (1,2). Glomerulopathy of membranous can also appear secondary to autoimmune diseases, infections, drugs and malignancies (1,2). iMN develops because of binding of a circulating antibody to an antigen that resides on podocytes (2,3). However, in secondary forms of membranous nephropathy (MN), the subepithelial deposits arose from circulating immune complexes with antigens that are originated from tumors, infectious agents or toxins and then implanted under the basement membrane (1-4). Patients with MN are presented with a broad range of urine protein excretion. However, the amount of proteinuria often fails to correlate with the quantity of immune deposits, demonstrable on fluorescent and electron microscopy (2-5). Currently, several therapeutic options are available, however, in some cases of iMN, spontaneous remission may happen too (2-5). Nonetheless, in a substantial number of patients the response to treatment is poor and the risk of progression to end stage renal disease is high. It was found that iMN may lead to end-stage kidney disease (ESRD) in 40–50% of adults patients in its long term course (2-5).The prediction of the disease intensely correlates with the remission rate (2-5). Patients age, gender, kidney function and proteinuria are predicting factors for progression to ESRD (3-5). However, some of those predictors may not be so relevant and does not predict the potential necessity to intensify the immunosuppressive (2-4). Interestingly, spontaneous remission may occur in up to 20–25% of patients with massive proteinuria (1-5). Therefore, prognostic markers in iMN would help clinicians to recognize potential candidates for rapid and robust intervention and implementing specific strategies. Generally, the development of biomarkers largely depends on the knowledge of the disease pathogenesis. In iMN, serologic diagnosis had been elusive because the target antigen was unidentified for several years. Then, it has become evident that binding of circulating autoantibodies to target antigens on the podocyte, will start the disease process (1-5). Up to now, a significant amount of efforts have been paid to identify the target antigens of iMN (2-5). Previously, studies of membranous nephropathy in a rat model namely, Heymann’s nephritis, proved that the subepithelial immune deposits containing of the target antigen, megalin, with circulating anti-megalin antibodies are formed in situ (1,3,6,10). However, megalin is not expressed on human podocytes and is not the target antigen in human disease (1-4). Despite the recent ground breaking discovery of the main podocyte antigen in iMN, Recently another new podocyte autoantigens have been detected and investigations is now focused on the development and authentication of a panel of antibodies to risk stratify patients and support clinical decision making.

Phospholipase A2 receptor, is normally expressed in podocyte membrane (1,5). In fact the phospholipase A2 receptor (PLA2R) is a type I transmembrane glycoprotein related to the C-type animal lectin family such as the mannose receptor. PLA2R regulates a number of biological responses produced by secretory phospholipase A2s (sPLA2s) (1,5,6). It was suggested that group of IB sPLA2/PLA2R pathway had a potential role in the production of pro-inflammatory cytokines. Also, PLA2R has been found to involve in the clearance of sPLA2s (5-7). Therefore, determining anti-PLA2r serum levels in patients with nephrotic syndrome should designate a probable diagnosis of iMN and in patients in which iMN had a pathology confirmation may be a determining factor to exclude secondary forms of the disease (1,2,5-8). Moreover, anti-PLA2r may be used as a marker of response to treatment (5-8). Thus, the recent finding on phospholipase-A2-receptor antibodies (PLA2R-Ab) may have a role in the progress of primary membranous glomerulonephritis and suggest the opportunity to measure a marker to help diagnosis, classify and finally monitor the course and outcome of patients with iMN (6-8).

However, the finding of autoantibodies to M-type phospholipase A2 receptor in iMN still evokes a question. Whether it is truly pathogenic? It is evident that iMN is an IgG4 dominant disease, (5-9). However IgG4 is not a complement antibody, this may debate on the pathogenic role of this antibody. Recent studies has suggested that PLA2R-IgG4 may bind mannose binding lectin, and thus activates complement via the mannose binding lectin pathway (5). Several investigators have also detected occurrence of anti-PLA2R antibodies in patients with secondary MN. Thus, more data are still needed before we can safely conclude that the presence of anti-PLA2R antibodies almost always reveals iMN and precludes the need to investigate for an underlying cause (5-10). Although the numbers of publications in this field are scares and still needs further investigation (6-10). Measurement of anti-PLA2R is now commercially available. We suggest storing serum samples at baseline and during follow-up. This would permit us to perform measurements at different time pointuntilall questions regarding the efficacy of anti-PLA2R antibody measuring in iMN needs full explanation (6-10).

We believe that, it is too early to abandon a kidney biopsy in patients with nephritic syndrome, and this newly introduced serologic study like another newly introduced measurements needs controversies, doubts and confirmations to finds its proper place in the field of clinical medicine.

## Author’s Contribution

MRA is the single author of this paper.

## Financial Disclosure

No financial support by any institution.

## Conflict of interests

None to declare.

## Ethical considerations

Ethical issues (including plagiarism, informed consent, misconduct, double publication and redundancy) have been completely observed by the authors.
